# Urinary tract infections and antibiotic use in pregnancy - qualitative analysis of online forum content

**DOI:** 10.1186/s12884-019-2451-z

**Published:** 2019-08-13

**Authors:** Flavia Ghouri, Amelia Hollywood, Kath Ryan

**Affiliations:** School of Pharmacy, University of Reading, Whiteknights, Reading, RG6 6UR UK

**Keywords:** Urinary tract infection, Prevention, Antibiotics, Online forum, Pregnancy, Pre-natal attachment, Antimicrobial resistance

## Abstract

**Background:**

Antibiotics are standard treatment for asymptomatic and symptomatic urinary tract infections (UTIs) in pregnancy. Their overuse, however, can contribute to antimicrobial resistance (AMR) and expose the foetus to drugs that might affect its development. Preventative behaviours are currently the best option to reduce incidences of UTIs and to avoid the use of antibiotics in pregnancy. The aim of this study was to explore women’s experiences of UTIs in pregnancy to develop an understanding of their concerns and to optimise and encourage behaviours that facilitate appropriate use of antibiotics.

**Methods:**

An online pregnancy forum in the United Kingdom (UK) was used to collect data on women’s discussions of UTIs. A total of 202 individual threads generated by 675 different usernames were selected for analysis. The data was organised using NVivo 11® software and then analysed qualitatively using inductive thematic analysis.

**Results:**

Women’s perceptions of UTIs and antibiotic use in pregnancy were driven by their pre-natal attachment to the foetus. UTIs were thought to be common and high risk in pregnancy, which meant that antibiotics were viewed as essential in the presence of suspected symptoms. The dominant view about antibiotics was that their use was safe and of little concern in pregnancy. Women reported an emotional reaction to developing a UTI. They coped by seeking information about behaviour change strategies to assist with recovery and through emotional support from the online forum.

**Conclusions:**

Women face dual risks when they experience UTIs; the risk from the infection and the risk from antibiotic treatment. Pre-natal attachment to the foetus is highlighted in the decision making process. The focus is on the shorter term risk from UTIs while undermining the longer term risks from antibiotic use, especially the risk of AMR. A balanced view needs to be presented, and evidence-based infection prevention strategies should be promoted, to women to ensure appropriate antibiotic use in pregnancy, to address the global challenge of AMR.

## Background

Pregnancy can increase the susceptibility of urinary tract infections (UTIs) in women because of physiological changes [1]. The vast majority of primary care antibiotic prescriptions issued to pregnant women in the UK are for UTIs [2] which suggests a high prevalence. Evidence from studies shows that asymptomatic infection alone can affect 2–12% of women [3]. The current management of UTIs in pregnancy is with a short course of antibiotics whether or not the infection is symptomatic. Asymptomatic bacteriuria (ASB) is diagnosed and treated through routine screening during the first trimester [4] which is in contrast to non-pregnant women where asymptomatic infections are not treated with antibiotics [5]. ASB is treated in pregnancy because studies have shown that bacterial colonisation of the urinary tract in pregnancy can cause adverse health outcomes e.g. there are risks of kidney infection, intra-uterine growth retardation and pre-term birth [6, 7]. The authors of a recent randomised control trial however have questioned the benefit of routine screening for ASB in the first trimester of pregnancy [8]. Kazemier et al. (2015) [8] found no association between ASB and growth retardation or pre-term birth and although an association was observed between ASB and kidney infection, the absolute risk was found to be low.

Excessive and unnecessary use of antibiotics is strongly associated with a rise in antimicrobial resistance (AMR) which is the ability of bacteria to survive in spite of antibiotic treatment leading to life threatening infections [9]. There is evidence from the UK and internationally which suggests that antibiotics to treat UTIs are overprescribed in pregnant women [10, 11]. Although AMR is a global public health threat to everyone, in pregnancy it can be particularly concerning due to the risk of resistant bacteria passing on to the neonate during birth which can be a vulnerable stage of life with regards to contracting infections. In addition to this, antibiotic use in pregnancy may also carry the risk of potentially teratogenic effects including spontaneous abortion [12] . Therefore, in light of AMR and the risk of adverse effects from the use of antibiotics, it is important that maternal antibiotic use is appropriate without compromising the health of women if they experience a UTI.

While a number of non-antibiotic options for UTI management have been studied, research has mostly focused on non-pregnant populations. A systematic review by the authors of the current study reported that preventative hygiene behaviour, such as washing the genitals after sexual intercourse, is the only evidence-based intervention linked to a reduced incidence of UTIs in pregnancy and therefore the most effective method of avoiding antibiotics [13]. Therefore women need to be encouraged, through effective communication, to adopt these preventative behaviours to minimise antibiotic use. Qualitative exploration of women’s perceptions can assist healthcare professionals by informing an in-depth understanding of their beliefs and concerns about experiencing UTIs during pregnancy. As this has not been researched before, this study aims to explore women’s perceptions about UTIs specifically during pregnancy.

## Method

### Design

Research has shown that searching for information online increases during pregnancy and women find online communities useful because of their accessibility [14, 15]. The website www.mumsnet.com was used to access naturally occurring data with regards to women’s perceptions about UTIs during pregnancy. Mumsnet is a popular parenting website in the UK and consists of conversational threads in a designated space called ‘*Talk’* where users have discussions on a wide array of topics. A Mumsnet census from 2009 showed that subscribers to the website are mostly white British women, 30–40 years old, with a degree qualification [16]. Although the demographic data is difficult to ascertain precisely and may have changed over the years, using the website as the medium for data collection provides access to naturalistic data where participants are open about their views due to the anonymous nature of posting on an online forum under a username. The data was analysed qualitatively using inductive thematic analysis [17].

### Procedure

Conversation threads on the website were searched using the search tool and limited to ‘thread title only’ under the topic of ‘pregnancy’. The search terms ‘urinary tract infection or UTI’, ‘cystitis’, ‘kidney infection’, ‘bladder infection’, ‘E.coli’, ‘antibiotic’, ‘antimicrobial resistance’, ‘amoxicillin’, ‘co-amoxiclav’, ‘ciprofloxacin’, ‘nitrofurantoin’ and ‘trimethoprim’ were used to extract comprehensive data about UTIs. The search was conducted between 01-01-12 to 30–11-17 to explore recent views. All relevant threads were selected and downloaded to a Microsoft Word® document and then organised using the qualitative analysis software, NVivo 11®. A total of 202 individual threads generated by 675 different usernames were downloaded and analysed as seen in Table 1.

**Table 1:** Number of threads per search term

Search terms	No. of threads
Urinary tract infection or UTI	121
Cystitis	18
Kidney infection	12
Bladder infection	4
Water infection	18
*E.coli*	0
Antibiotic	19
Antimicrobial resistance	0
Amoxicillin	2
Ciprofloxacin	0
Co-amoxiclav	0
Nitrofurantoin	4
Trimethoprim	4
Total	202

### Data analysis

The data was analysed using inductive thematic analysis, using the guide recommended by Braun and Clarke (2006). Thematic analysis is a method used to analyse qualitative data that provides flexibility in identifying, analysing and reporting patterns in data. Inductive thematic analysis is directed by the content of the data and was chosen to allow collective exploration and interpretation of women’s perceptions of UTIs in pregnancy. The data was read by FG multiple times to achieve familiarisation and to generate detailed codes. Codes were then organised and developed by FG into themes by examining and reflecting on broader patterns in the data. Themes were reviewed by all authors and refined by referencing back to the data. A thematic map was generated which shows how the themes are linked. Illustrative quotes from the data have been used to evidence each theme. Quotes have been edited for clarification where appropriate.

### Ethical consideration

Mumsnet is a website that provides a forum for discussions where users are required to create a username to post a comment and they are informed that their posts are visible to anyone on the internet. The terms of use of www.mumsnet.com explicitly state that the website and its content are copyright to Mumsnet and any submission of data by users can be edited or published by Mumsnet for any purpose, commercial or otherwise, that the website considers appropriate. Therefore Mumsnet was contacted to explore whether they deemed this study an appropriate use of their data. Mumsnet confirmed approval and gave permission for the researchers to download the data to conduct the study. Individual users could not be informed about the study or contacted for explicit consent to use the content that they have posted on the website as users post under a username and no contact details are provided. To ensure scientific rigour ethical approval was sought and obtained from the University of Reading’s Research and Ethics Committee (Ref 17/30).

Public attitudes with regards to conducting research using social media are conflicting [18]. The rationale behind proceeding with the research was that data was not collected from a restricted space but an online open public forum accessible to anyone with an internet connection. There was a low risk of harm or distress because users had voluntarily shared their views. No identifiable information was collected or analysed during the conduct of this study protecting the identity and confidentiality of users. Users on the website posted using fictional usernames which were further changed to pseudonyms to anonymise quotes as an additional precaution to protect anonymity and confidentiality. Upon balancing the harms and benefits of this study, the authors believe that the study meets the British Psychological Society’s guidelines for internet-mediated research [19] and the Association of Internet Researchers’ recommendations for ethical decision making and internet research [20]. Exploring and voicing the experiences of women would lead to better understanding of healthcare needs by healthcare professionals and benefit women during pregnancy thus justifying this research ethically.

## Results

**Fig. 1:**
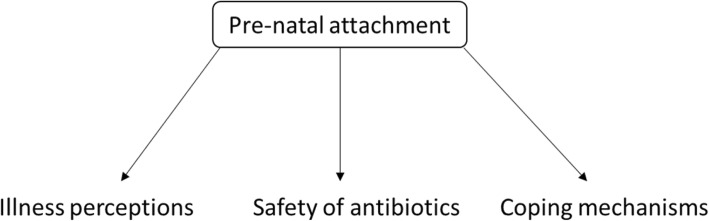
Thematic map

Analysis of the data led to construction of three subthemes and an overarching theme as shown in Fig. 1. The primary theme relates to women’s pre-natal attachment to the foetus which is reflected in the subthemes that describe women’s perceptions of UTIs in pregnancy, the safety of antibiotics and coping mechanisms employed to deal with the impact of the illness. The themes with illustrative quotes are described below.

### Pre-natal attachment

The primary overarching theme describes the pre-natal attachment or bond that women feel towards the foetus during pregnancy. Cranley (1981, pg. 282) described prenatal attachment as ‘the extent to which women engage in behaviours that represent an affiliation and interaction with their unborn child’ [21]. Analysis of the data in this study strongly demonstrated women’s attachment to their baby which was visible in the types of questions they asked each other and the language that they used in the discussion on the forum. The findings also show that while women discussed a range of issues, the main concern for most, with regards to experiencing a UTI, was concern for their baby.

I’m just worried for my little nugget, think I’ll ask to check the heartbeat when I go back for peace of mind. (Luna).

Hi I’m 6 + 4 and had a scan today…got to see a little heartbeat: -) only problem was traces of blood in my urine so they gave me antibiotics for what they suspect is a UTI…just worried now after seeing the heartbeat that taking the antibiotics will do something to baba?? (Sally).

The following section describes women’s perceptions of UTIs and antibiotics in pregnancy and shows how pre-natal attachment is reflected in the three subthemes.

### Illness perceptions

The risks of UTIs were viewed in terms of how the infection could impact the mother’s health e.g. developing a kidney infection, and the risks to the pregnancy in terms of effects on the foetus e.g. pre-term birth or miscarriage. The majority of discussions, however, focused on the impact on the foetus.

I’m really worried as I’m aware UTIs if left untreated can cause miscarriage, I feel like a sitting duck! I’m obviously glugging away at the water and cranberry juice but the pains are worrying me. (Rachel).

Untreated UTIs can lead to permanent kidney damage for you and premature labour. I’m not trying to scare you and I’m sure you’ll be fine but could you get in touch with your MW [midwife] and get their opinion. (Jane).

For most women it was the diagnosis of a UTI rather than the difficult symptoms per se that made them anxious, as even those who had an asymptomatic infection shared similar concerns about the UTI impacting pregnancy and harming the foetus.

Had no symptoms but told I had a bad UTI, I’m now absolutely petrified that I’ve found out too late and my baby will be harmed (Shadane).

Most women also viewed UTIs as being very common in pregnancy. As Stella described, “Never had one outside of pregnancy, you are more prone to them unfortunately!” In fact, it was thought that pregnancy caused UTIs to the extent that any troubling symptom could be the sign of a UTI.

“I always find in pregnancy that everything is put down to a UTI.” (Linda).

At the same time UTIs in pregnancy were also viewed as harder to diagnose and more difficult to treat compared to when not pregnant. The reasons were thought to be due to an overlap between normal pregnancy symptoms and those characteristic of UTIs. For example, as Jane indicated, “symptoms of UTI are quite difficult in pregnancy as you have a lot of them anyway”. Biochemical changes in the body due to pregnancy were also attributed to making diagnosis more difficult.

I also had trouble getting diagnosed when pregnant and was told by the midwife that it’s because there are so many things present in your pee and altering what’s in your pee when pregnant that it can be hard to get a dip result indicating a UTI. (Bella).

Ultimately, a desire to protect their baby, arising from pre-natal attachment, led most women to view UTIs to be so risky that urgent treatment with antibiotics was considered an absolute necessity and delaying or ignoring any symptoms was deemed “irresponsible”.

I’m surprised they haven’t given you any antibiotics straight away as it can cause early labour or a small baby if left untreated. Mine’s been in my kidneys, the pain has been horrendous. Don’t suffer if you need to ring and ask for antibiotics. (Aria).

If you weren’t pregnant you could maybe take your friend’s advice to drink water and cranberry juice and wait it out, but given that you are pregnant it’s irresponsible advice to be honest. (Nikita).

In summary, the majority of women perceived UTIs to be more dangerous in pregnancy compared to when not pregnant due to the risk of serious consequences such as miscarriage or pre-term birth. They also considered UTIs to be a fairly common occurrence with diagnosis and treatment of the infection more problematic in pregnancy. Pre-natal attachment to the foetus meant most women considered antibiotics to be absolutely essential for treatment and avoiding them, or a delay in seeking help was seen as irresponsible behaviour.

### Safety of antibiotics

A few women expressed reluctance and questioned the use of antibiotics in pregnancy for the treatment of UTIs. The reasons varied from concerns about teratogenicity, effects on long term immunity or personally experiencing antibiotic side effects. For example, Liza was fearful about the effects on the foetus, “I’m petrified that taking amoxicillin will harm baby!” while another website user was concerned about longer term effects such as the development of allergies in the child.

Personally I would be wary of it [antibiotic] because there is a link between taking antibiotics in late stage pregnancy and the baby having eczema and allergies. I took antibiotics in late pregnancy and my daughter has multiple food allergies and eczema (Tania).

In spite of these concerns, which were expressed by only a small proportion of women, most considered antibiotics to be generally safe.

Antibiotics are one of the few things they are really sure about giving to pregnant women, precisely because we get infections. (Tula).

The majority of women compared the risks of a UTI with the risks of using antibiotics and viewed antibiotics as the safest option for normal progression of their pregnancy. They drew on their personal experiences of using antibiotics or viewed a prescription as proof that antibiotics were not dangerous. As Carey suggested, *“*Doc wouldn’t prescribe if dangerous. It’s more dangerous to leave a UTI, as at its worst it can cause kidney issues and miscarriage”. This view also meant that some women thought that it was better to take antibiotics “just in case”.

The doc said it wouldn’t do me any harm and better than not taking them just in case I had needed them (if that makes sense). I don’t think you should feel guilty as the doc will have given you antibiotics that wouldn’t affect your baby. (Usha).

Thus when discussing the safety of using antibiotics to treat UTIs, whether or not they viewed them as safe in pregnancy, women’s primary motivation was to protect the foetus owing to pre-natal attachment. For a small proportion of women it was the uncertainty of how the antibiotic might affect the foetus or their child’s immunity in the long term that led them to be wary but for the remaining majority, antibiotics were perceived as the safest and most effective management option.

### Coping mechanisms

Pregnancy can understandably be an emotional time and unsurprisingly women on the forum described it in similar terms. Experiencing painful and frustrating symptoms of a UTI, coupled with a fear of how the infection might impact the pregnancy, had a considerably enhanced emotional impact on many women. Throughout the data, highly emotive language featured quite strongly to express discomfort and frustration. For example,

I just really worry about taking things when pregnant and feel so emotional atm [at the moment]!! Tia [thank you in advance] x (Serena).

One particular quote highlights strong feelings of guilt alongside the frustration,

I don’t know why but it makes me feel like such a failure each time the results come back with an infection still present. I get angry with myself that I can’t get my body to do its job to fight it and I’m putting my baby at risk. Stupid I know but I can’t help it xx (Nadia).

The majority of women therefore used the forum as a way of coping via two main functions; by using the forum for emotional support or by seeking information and advice. Some women coped by expressing their thoughts and feelings and seeking people with similar experiences whereas others sought advice on the forum about measures they could take to ease their symptoms and clear the UTI.

Using the forum as an online social support system to cope emotionally was seen throughout the data. For example, as one person posted, “Not sure why I’m posting, may just need a bit of hand holding …” (Hope) and another person while consoling someone stated, “It’s such a lonely illness try [to] get some company or people to talk [to] on the phone/text or Mumsnet of course” (Rachel). The emotional impact of the illness was particularly strong in women who experienced recurrent or resistant infections requiring multiple courses of antibiotics. They expressed frustration and feeling a lack of control as emphasised in the exchange below.

Now dreading getting another UTI as it will prove difficult to treat according to my GP. Only 19 weeks along, so this better not happen again (Veda).

I feel your pain - aside from the physical symptoms it was the “how will I ever get rid of this without Abs [antibiotics]?” question that really dragged me down (Response by Lynn).

As mentioned above, many women also used the forum to seek and give advice on measures to cope actively with their illness, especially if they experienced recurrent infections. Preventative measures such as drinking cranberry juice, using over-the-counter (OTC) cystitis relief remedies or following certain hygiene behaviours, like wiping the genitals from front to back etc., were some of the measures that women discussed. Perceptions around these measures, however, were varied and the suitability of some of the remedies was also often questioned. For example, one woman advised avoiding cranberry juice while another found it be effective and a better option than antibiotics especially for milder infections.

As much water as you can drink. Mix some bicarb in water and have that if you have some, tastes awful but helps. Avoid cranberry juice. Avoid caffeine. I am a UTI veteran! (Alia).

Cranberry juice is good as well. If it is mild this may flush the infection out and is gentler than antibiotics which will be likely to cause thrush… (Marina).

Similarly, there were differing views regarding the suitability of over-the-counter cystitis relief treatment.

I can’t have the sachets I normally drink to ease symptoms as they are unsuitable for pregnant women (Delia).

My doctor said it was safe to use the cystitis relief sachets along with drinking plenty of water and cranberry juice and apparently it should go within 48 h (Irene).

To summarise, UTIs in pregnancy had an emotional impact on most women which left them feeling frustrated at a time when they were already going through physical changes in their bodies. They used the online forum to find ways to cope either through exchange of information and advice or for emotional support. The discussions, both when exchanging tips and when venting emotionally, centred significantly on the impact on the pregnancy which again highlights women’s pre-natal attachment to the foetus.

## Discussion

This study explored women’s perceptions of experiencing a UTI during pregnancy as discussed on an online forum. The results indicate that women view UTIs in pregnancy primarily from the lens of being an expectant mother and pre-natal attachment to their unborn baby drives them to put the safety of the foetus at the very core of how they view the illness and how they behave at the onset of a UTI. Pre-natal attachment is a theoretical construct drawn from John Bowlby’s theory of Human Attachment [22]. Its relevance to antenatal care lies in the fact that it is useful in motivating women to adopt practices that facilitate good health for themselves and their unborn child [23].

Previous studies [24, 25] exploring women’s perceptions of UTIs report that the condition can significantly affect women’s quality of life. The current study supports these findings, as described in the first subtheme relating to illness perceptions, and highlights how the state of being pregnant means that women view UTIs to be more common and linked to serious consequences compared to when not pregnant. The high risk perception and the view that UTIs are common in pregnancy encourages women to seek antibiotic treatment at the onset of any probable symptom even though it may be a normal pregnancy occurrence such as increased frequency of urination. The second subtheme, which relates to the safety of antibiotics, highlights that apart from a small proportion, most women on the forum favour antibiotic treatment which is in contrast to a study conducted with non-pregnant women [26]. The perception that antibiotics are generally safe in pregnancy is also unusual in light of other studies that report that women view antibiotics to be moderately harmful [27] and that women overestimate teratogenic risk from exposure to medicines [28]. The last subtheme, which describes coping mechanisms, highlights the negative impact that UTIs can have on women and how they cope by adopting two distinct approaches. These approaches are seeking advice on actions to take to cope with the UTI or using the forum for emotional support. Both of these approaches are reflected in health psychology literature and correspond to problem-focused or emotion-focused coping respectively which are two styles that people might adopt to cope with stressors [29].

### Implications of study

The findings from this study have implications for how women should be encouraged to take care of their health during pregnancy with regards to urinary infections and the use of antibiotics. The data highlights how pregnant women are faced with dual causes of risk when they experience UTIs; the risks arising from the infection and the risks from using antibiotics. This duality of risks leads most women to perform a cost-benefit analysis and pre-natal attachment means they prioritise safeguarding the foetus against the short term risks of a UTI while overlooking the longer term risk of AMR from using antibiotics. This might lead to increased demand and overuse of antibiotics and contribute to the problem of AMR, which can affect not just the foetus but women themselves and society at large.

Current data on the incidence and outcomes of UTIs in pregnancy in the UK along with improved diagnostics is needed to ensure antibiotics are not being overused. As well as this, women need balanced information from healthcare professionals not only about the risks of UTIs but also of excessive antibiotic use. Future research can investigate the usefulness of emphasising pre-natal attachment in interventions designed to promote responsible use of antibiotics.

Although pregnancy can increase women’s susceptibility to a UTI, prevention measures can protect against it [13, 30, 31]. Information about prevention through hygiene behaviours needs to be standardised and emphasised as the best way of preventing UTIs in pregnancy. In addition, the perception that pregnancy causes UTIs, as seen in this study and promoted by the National Health Service [32], requires challenging as it reflects a medical model of illness that attributes the cause of illness to external factors beyond individual control. Instead, women should be encouraged to use a problem-focused coping style through the adoption of preventative hygiene behaviours so that they can appreciate the controllability of this illness rather than rely solely on a medical solution i.e. antibiotics. Promoting such a behavioural model of illness, which sees the individual’s behaviour as the solution to a health problem, has also been reported to lead to practise of healthier behaviours and outcomes that are sustained over a long period of time [33]. Lastly, the emotion-focused coping seen on the forum highlights women’s need for emotional support and a sensitive approach to their care during pregnancy in relation to UTIs.

### Strengths and limitations of study

The strengths of this study lie mainly in the method used for data collection. The advantage of using an online forum to collect data was that it provided access to a wide range of participants across the UK. Online forum postings increase the perceived sense of anonymity in participants, which can increase disclosure compared to face-to-face interviews. Data was also immediately available for analysis and circumvented transcription errors arising from interviews.

Using an online forum, however, also contributed to the limitations of this study. Women used descriptive text and emoticons to express their feelings, but using an online forum could result in a loss of insight that facial expressions or verbal tone can offer to exploring perceptions. It was difficult to characterise the exact demographics of website users and only views of women who had access to the internet and had subscribed to the forum could be analysed. Different cultural groups in the UK may also have different norms of behaviour in pregnancy and the views of women from varying backgrounds may differ from what was captured from the forum. Therefore, the interpretation and transferability of the results should be made within this context and broad generalisations may not be appropriate.

## Conclusion

UTIs are prevalent in pregnancy and can cause women considerable stress and anxiety. Their primary concern stems from how the infection or the antibiotics might affect their baby. Although some women question the safety of antibiotics, most women adopt a risk appraising process which leads them to regard antibiotics as absolutely essential and safe for use in pregnancy if they experience any suspected symptom of a UTI. Pre-natal attachment may cause women to focus solely on the risks of a UTI while under-appreciating the risks of antibiotics, particularly the threat from AMR, which is a major global challenge.

## Data Availability

All datasets supporting the conclusions of this article were collected from www.mumsnet.com and are available from the corresponding author on reasonable request.

## Abbreviations

AMR: Antimicrobial resistance
ASB: Asymptomatic bacteriuria
*E.coli*: *Escherichia coli*
UTI: Urinary tract infection
